# Partial Splenectomy for Splenic Cyst using a Bipolar Radiofrequency Device

**DOI:** 10.4021/gr2009.07.1301

**Published:** 2009-07-20

**Authors:** Hieronymus PAM Poos, Deepu Daryanani, Joost M Klaase

**Affiliations:** a Department of Surgery, Medisch Spectrum Twente, the Netherlands

**Keywords:** Partial splenectomy, Splenic cyst, Bipolar radiofrequency device

## Abstract

The main goals of spleen preserving surgery are control of peroperative bleeding and maintaining the spleen’s function postoperatively. Several techniques of spleen preserving surgery have been described. This report presents a new technique to perform partial splenectomy. We performed this partial splenectomy with a bipolar radiofrequency (RF) device in a 21 years old woman with a splenic cyst, with almost no peroperative blood loss.

## Introduction

The incidence of splenic cysts is low. A difference is made between primary cysts which are congenital or parasitic (rare in Western countries) and secondary cysts, being posttraumatic in origin or due to splenic infarctions or infections [1].

Partial splenectomy for benign disease of the spleen has an immunologic advantage above total splenectomy, because preserving a part of the spleen prevents the risk of a perilous postsplenectomy sepsis [2-4]. Nevertheless there is a significant risk of peroperative bleeding because of the spleen’s anatomy. Various techniques have been described for partial splenectomy such as suture control, compression of the spleen [5], argon beam coagulator [6], stapling techniques [7], cystectomy/marsupialisation [8] or using a Lin’s clamp[9]. However in order to prevent bleeding from the cut surface all of them require clamping of the splenic hilum. Partial splenectomy has been described using a unipolar “cool tip” radiofrequency (RF) [10] device or unipolar RF technology combined with adequate irrigation with saline [11].

The current report presents a technique for spleen preserving surgery using a new disposable bipolar RF device developed for liver resection by professor Habib from the Imperial college in London.

## Case Report

### Case

**Figure 1:**
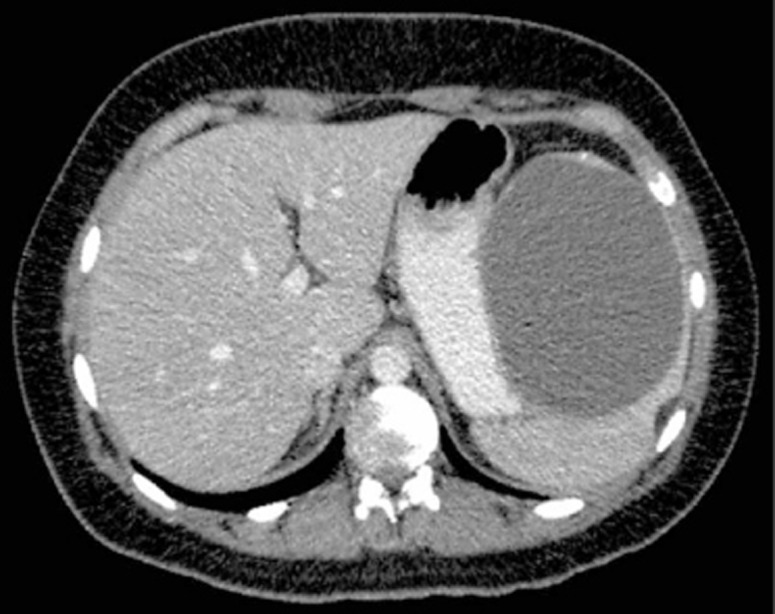
Computed tomography scan showed a cyst (11 × 8.5 cm) in the upper part of the spleen.

A 21 years old woman, with no medical history (especially no trauma or signs of infection) presented with nagging pain in the upper left abdomen. Ultrasound showed a large cyst, of which the origin was not clear. Subsequently computed tomography scanning showed a cyst localized in the upper part of the spleen, 11 × 8.5 cm in size (Fig 1). There was no retroperitoneal or mesenterial lymphadenopathy.

### Surgical technique

**Figure 2:**
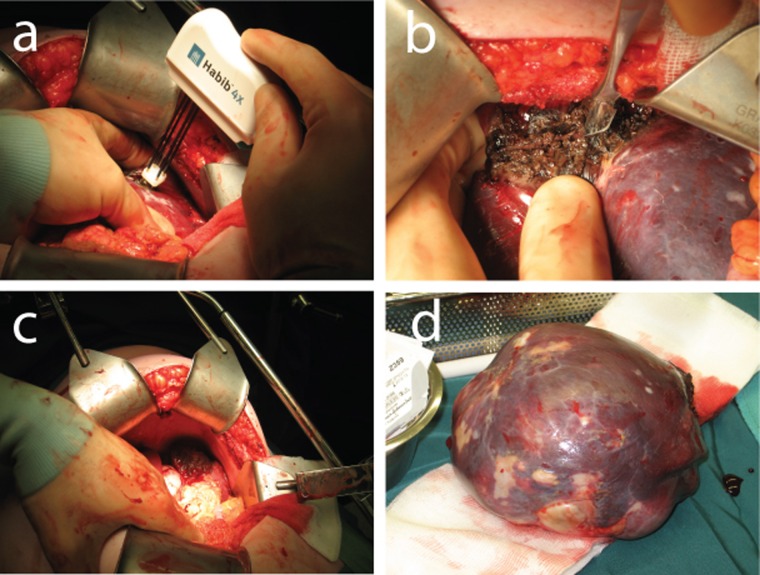
(a) Coagulation of the spleen with the Habib sealer. (b) Transection of the upper part of the spleen. (c) The preserved inferior part of the spleen and bloodless resection surface. (d) Resected splenic cyst (epidermoid cyst).

Under general anaesthesia a left subcostal laparotomy was performed with the patient in supine position. The spleen was fully mobilized. The technique used for this partial splenectomy was similar to that described for liver resection. In brief, after mobilization of the spleen, an intended line for division of tissue was first drawn on the spleen capsule by electrocautery, 1 cm away from the cyst. Using the disposable bipolar Habib 4X sealer (Habib™ products, Angiodynamics, Habib 4X Open surgery bipolar resection device) the upper part of the spleen including the cyst was coagulated along the resection line introducing the sealer perpendicularly into the spleen (Fig 2a). The probe is introduced adjacent to the last coagulated area, in a serial fashion, until the area to be transected is ablated. The power of the RF generator was set at 50 watts. Subsequently a scalpel is applied to divide the parenchyma through the coagulated area (Fig 2b). The resection was performed without vascular clamping of the splenic hilum and could be completed with almost no blood loss (Fig 2c, d). The duration of ablation was 20 minutes.

### Postoperative course

The postoperative course was prosperous with a minimal decrease in haemoglobin and there was no need for blood transfusion. The patient was discharged home on the 6th postoperative day and 3 weeks post surgery the patient was functioning normally without any complaints. The histopathologic diagnosis was an epidermoid cyst.

## Discussion

Partial splenectomy has an immunologic advantage above total splenectomy. However there is a risk for peroperative bleeding. When a safe operative technique is used, this has to be the treatment of first choice in benign splenic disease.

Radiofrequency ablation seems a useful technique to perform partial splenectomy [10, 11]. Moreover because other known techniques (e.g. suture control, compression of the spleen [5], argon beam coagulator [6], stapling techniques [7], cystectomy/marsupialisation [8] or using a Lin’s clamp [9]) require clamping of the spleens hilus. An unipolar device mentioned before needs the use of 4 patient return electrodes in each procedure which has to be positioned on well vascularised muscle mass of the patient. This has to be done to remove the current safely from the patient. A return electrode burn can occur when the heat produced, over time, is not safely dissipated by the size or conductivity of the patient return electrode [12-13]. A bipolar device does not need return electrodes. Also, when treating patients with an implanted cardiac device, there is a risk of electromagnetic interference when using a unipolar device. A bipolar radiofrequency device, which was used in the present case report, lacks this risk [14].

At present, partial splenectomy with the disposable bipolar Habib 4X sealer seems to be the most simple and safest method to perform spleen preserving surgery.
